# Hypoxic Stress-Induced Tumor and Immune Plasticity, Suppression, and Impact on Tumor Heterogeneity

**DOI:** 10.3389/fimmu.2017.01625

**Published:** 2017-11-24

**Authors:** Stéphane Terry, Stéphanie Buart, Salem Chouaib

**Affiliations:** ^1^INSERM UMR 1186, Integrative Tumor Immunology and Genetic Oncology, Gustave Roussy, EPHE, Fac. de médecine – Univ. Paris-Sud, University Paris-Saclay, Villejuif, France

**Keywords:** phenotypic plasticity, tumor microenvironment, hypoxia, antitumor immunity, myeloid-derived suppressor cell, epithelial–mesenchymal transition, NK, T cells

## Abstract

The microenvironment of a developing tumor is composed of proliferating cancer cells, blood vessels, stromal cells, infiltrating inflammatory cells, and a variety of associated tissue cells. The crosstalk between stromal cells and malignant cells within this environment crucially determines the fate of tumor progression, its hostility, and heterogeneity. It is widely accepted that hypoxic stresses occur in most solid tumors. Moreover, cancer cells found within hypoxic regions are presumed to represent the most aggressive and therapy-resistant fractions of the tumor. Here, we review evidence that hypoxia regulates cell plasticity, resistance to cell-mediated cytotoxicity, and immune suppression. Exposure to hypoxia occurs as a consequence of insufficient blood supply. Hypoxic cells activate a number of adaptive responses coordinated by various cellular pathways. Accumulating data also suggest that hypoxic stress in the tumor microenvironment promotes tumor escape mechanisms through the emergence of immune-resistant tumor variants and immune suppression. Thus, solid tumors seem to build up a hostile hypoxic microenvironment that hampers cell-mediated immunity and dampen the efficacy of the immune response.

## Introduction

**Figure 1:**
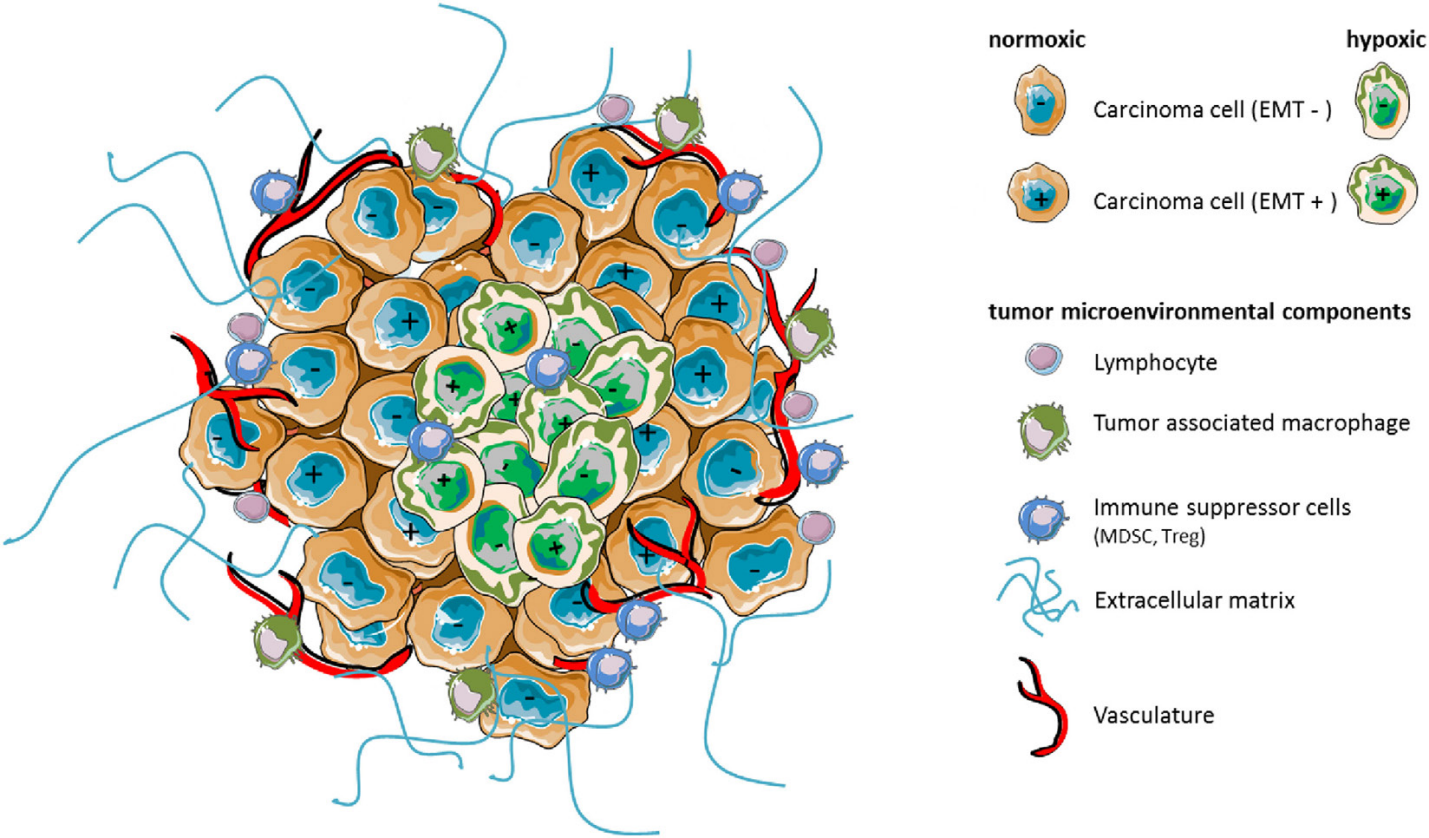
Hypoxia as a major component of the tumor microenvironment shapes stroma reactivity and tumor heterogeneity. Tumors contain distinct cell types that collectively enable tumor growth and progression. Multiple stromal cell types, thus, create microenvironmental conditions that change as tumor is growing. In particular, hypoxia caused by insufficient concentration of oxygen can affect the phenotype (e.g., epithelial EMT− vs mesenchymal EMT+) of certain carcinoma cells leading potentially to increased heterogeneity of the cancer cell population, intrinsic resistance to antitumor immunity, or immunosuppression by affecting the functions, the phenotype, and/or the number of surrounding microenvironmental cell components.

The tumor microenvironment (TME) is a complex system that contains numerous cell types playing important roles in tumor development and progression. In such system, hypoxia appears as an essential metabolic element that may help to shape cellular plasticity and tumor heterogeneity (1). Hypoxia is characterized by lack of O_2_ in a setting where hypoxic tissues are inadequately oxygenated (2). Thus, in cancer, abnormal formation of the vasculature in rapidly growing tumor mass results in heterogeneously distributed areas of low oxygen pressure, generating hypoxic stress. Both non-cancerous and cancer cells adapt to the hypoxic microenvironment by regulating the hypoxia-inducible factor (HIF) family of transcription factors. HIFs are dimeric proteins composed of an O_2_-sensitive α subunit (HIF-1α, HIF-2α, or HIF-3α) and a β subunit (HIF-2β). Under hypoxic stress, hypoxia-dependent stabilization of HIF dimers allows for the induction of numerous genes regulating biological processes and functions in cells, including angiogenesis, cell survival, proliferation, pH regulation, and metabolism. Accumulating evidence also points to hypoxia as an important trigger for cancer cell invasion or metastases *via* the activation of hypoxic cascades and HIF-1α. This could at least partly explain associations previously found in human tumors between hypoxic stress and tumor progression, or other adverse effects (3). Moreover, cancer cell adaptation under hypoxia allows for their survival, maintenance of cancer stem cells (CSCs) (1), and might give rise to heterogeneity and the emergence of therapy-resistant phenotypes (Figure 1), with implications for chemo- and radio-resistance, as well as resistance to the immune system. The intent of this minireview is to present recent evidence suggesting that hypoxia influences cancer cell plasticity and cell phenotype that may have consequences on immune resistance and immune suppression in the TME.

## Hypoxia Induces Resistance to Cell-Mediated Cytotoxicity

**Figure 2:**
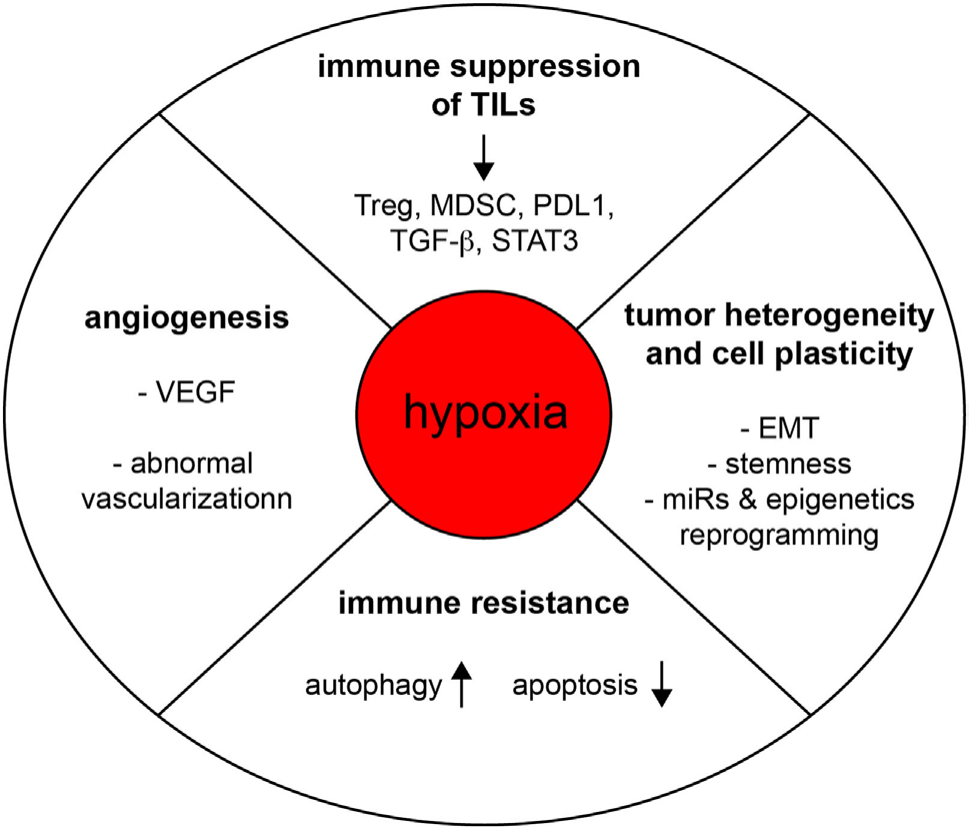
Hypoxia subverts the function of the immune system and promotes tumorigenesis. Hypoxia regulates tumor progression through various mechanisms of action, including the promotion of angiogenesis, tumor heterogeneity, cell plasticity, immune resistance, and intratumoral immune suppression.

Recent advances in immunotherapy approaches have largely improved the survival of many patients with advanced malignancies (Figure 2). However, the high prevalence of non-responders also reminds us that we possess only a partial understanding of the mechanisms at play in the immune resistance of tumors. Cancer immunotherapy approaches generally aim to induce a strong cytotoxic T lymphocyte (CTL) response, with the prevailing view that the generation of a sufficiently high frequency of CTL response will result in a clinically significant regression of tumor burden. Yet, even as interest for immunotherapy has grown, it becomes increasingly apparent that tumors can efficiently evade or inactivate even substantial immune responses. This is accomplished through establishing a metabolically hostile microenvironment, by denying T cells access to the tumor, and through selection of immune-resistant cancer cell variants. We previously showed that hypoxia may induce tumor resistance to CTL-induced killing through mechanisms involving increased phospho-STAT3 in target cells (4) or hypoxia-induced autophagy (5). HIF-regulated miR-210 could also be an important mediator of susceptibility to autologous CTL-mediated lysis (6). Interestingly, targeting autophagy in hypoxic melanoma B16-F10-engrafted tumors, improved the efficacy of a TRP-2-peptide cancer vaccine leading tumor regression *in vivo* (4). Previous work also showed that hypoxia could increase ADAM10 expression, with concomitant decrease surface expression of MICA leading to cancer cell resistance to cell-mediated lysis by innate immune effectors such as NK cells (7). Furthermore, activated autophagy under hypoxia has been proposed as a potent mechanism of tumor escape to NK-mediated immune surveillance (8, 9). Indeed, in hypoxic cancer cells, Granzyme B seems to be selectively degraded upon hypoxia-induced autophagy, thereby inhibiting NK-mediated cell lysis (9). The role of autophagy in regulating NK-mediated immune responses was also investigated in a clear cell renal cell carcinoma model harboring mutation in von Hippel–Lindau gene which stabilizes HIF2, and correlating with resistance to NK-mediated killing (10). We observed that inositol 1,4,5-triphosphate receptor type 1 (ITPR1), an endoplasmic reticulum Ca^2+^-release channel involved in authophagy (11), was overexpressed in mutated cells in a HIF2-dependent manner, while protecting the cells against NK cells attacks by inducing autophagy (10).

## Hypoxia Plays a Key Role in Regulating Immune Suppression

Hypoxia has been associated with potentiation of immunosuppression, *via* its role on the activation of an immune suppressive network (Figure 2). This has been reviewed in detail elsewhere (12, 13). The effect of hypoxia on intratumoral T CD8 T cells has not yet been fully elucidated (12). Dendritic cells differentiation and maturation seem to be inhibited under hypoxia, distracting them from their T-cell activating functions (14). In a setting where immunosuppression mediated by regulatory T cell (Treg) could represent one relevant mechanism for tumor immune evasion, work by Facciabene and colleagues nicely showed that tumor hypoxia promotes the recruitment of Tregs through inducing chemokine CC-chemokine ligand 28 (CCL28) expression, which, in turn, promotes angiogenesis and tumor tolerance (15). Nonetheless, a direct role of hypoxia in regulating Treg functions deserves further investigations in order to clarify some data discrepancies (16, 17).

Recently, hypoxia-induced Nanog was investigated for a potential role in immune suppression. Nanog is a stemness-associated transcription factor, and is selectively induced in hypoxic areas of B16-F10 tumors (18). Targeting of Nanog in this model system significantly reduced immunosuppressive cells (Treg cells and macrophages) while increasing CD8^+^ T effector cells in the tumor bed in a manner that seems dependent, at least in part, on TGF-β1 production (18). Thus, Nanog appears to regulate TGF-β1 expression by directly binding the mouse TGF-β1 proximal promoter. In addition to addressing a major role of Nanog in hypoxia-driven immunosuppression, these findings also pointed out a novel mechanistic link between hypoxia-induced Nanog and regulation of TGF-β1.

Tumor-infiltrating myeloid cells, including myeloid-derived suppressor cells (MDSCs) and tumor-associated macrophages (TAMs), represent important components of the hypoxic TME likewise known to contribute to tumor-mediated immune escape. In addition to studies reporting a role of HIF-1 and HIF-2 in the promotion of macrophage angiogenic property (19), HIF-1α could also contribute to macrophage-mediated inhibition of T cells (13, 20). Gabrilovich et al. have elegantly shown that hypoxia *via* HIF-1α impedes the function of MDSC in the TME and redirects their differentiation toward TAMs, further providing relevant clues to how different myeloid suppressive cells may cooperate to immune suppression in the TME (21). Recently, we showed that expression of programmed death-ligand 1 (PD-L1) by tumoral MDSCs is upregulated under hypoxia resulting in increased MDSC-mediated T cell tolerance (22). In fact, HIF-1α regulates the expression of PD-L1 by binding directly to a hypoxia response element in the PD-L1 proximal promoter. Moreover, a significantly higher expression of PD-L1 was found on tumor-infiltrating MDSCs than on splenic MDSCs isolated from different tumor-bearing mice. Of importance, PD-L1 can also be induced in cancer cells exposed to hypoxia (22, 23).

## Hypoxia, Tumor Plasticity, and Heterogeneity

The underlying biology of tumor heterogeneity has remained a conundrum for scientists and clinicians alike (Figure 2). Yet, there is now a consensus among researchers in support of the idea that some cancer cells have the capacity to transit between epithelial and mesenchymal phenotypes, or states, *via* epithelial–mesenchymal transition (EMT) or the reverse process, mesenchymal–epithelial transition (MET) (24). Since the cells may switch back and forth along the EMT spectrum, these reversible cell state transitions also reflect the so-called plasticity of cancer cells, and as such may represent an important source of phenotypic heterogeneity in the tumor. Cell plasticity seems to be tightly regulated by contextual signals from the TME, vasculature, and anatomic sites. For instance, EMT of carcinoma cells may be induced by the local microenvironment and hypoxia (25). TGF-β, which can be induced and activated under hypoxic conditions might exert important functions in this setting. HIFs and HIF-regulated genes have been associated with marked CSC properties in various cellular contexts including Glioma (26), hematological malignancies (27), breast cancer cells (28), or in circulating tumor cells (CTCs) derived from breast carcinoma MDA-MB-231 engrafted tumors (29). It is noteworthy to add that cancer cells are exposed to chronic or intermittent hypoxic stresses, and depending their location, to various hypoxia levels. This should also contribute to increasing the intratumoral heterogeneity in space and time. In addition, there are also epigenetics determinants involved in cell plasticity (24). Recently, the possibility has emerged that certain cancer cells and cancer clones might use cell plasticity and phenotypic switching to escape immune attacks, and various therapeutic intervention, including immunotherapies (30–32). Using a model of lung adenocarcinoma cells, we have recently proposed that the transition toward a more mesenchymal phenotype under hypoxia may occur only in a fraction of hypoxia-exposed cancer cells (33), and that carcinoma cells with a more mesenchymal state were functionally more resistant to CTL- and NK-cell-mediated lysis compared to their more epithelial counterparts, which may lead to the emergence of immunoresistant variants (33). In this line, Ricciardi and colleagues observed that exposure to inflammatory cytokines can endow cancer cells undergoing EMT with a number of immunomodulatory effects, including interference with proliferation, differentiation, and apoptosis of NK, T, and B cell populations (34). Hypoxia-induced EMT could also promote an immunosuppressive TME as shown by Yu et al. who reported that hypoxia-induced EMT in hepatocellular carcinoma cells promotes an immunosuppressive TME by increasing expression of CCL20, which acts on monocyte-derived macrophages, eventually promoting metastasis (35). Another intriguing observation is that HIF-1 can stimulate CD47 expression, an important factor for maintaining CSCs, that likewise enables breast cancer cells to avoid phagocytosis by macrophages (36). Future investigations should address molecular paradigms linking hypoxia, cell plasticity, and CD47-mediated resistance to phagocytosis. Moreover, in syngeneic immunocompetent mouse tumor models, CD47 was found to promote evasion from T-cell responses (37).

## Conclusion

Despite the recent success in the field of immunotherapy, it has become clear that the induction of a good T-cell response is not efficient to control tumor progression and that simply avoiding immune suppression does not necessarily result in the induction of an effective antitumor immune response. Converging evidence suggests that tumor cell plasticity may lead to the emergence of immunoresistant variants. Therefore, we argue that targeting carcinoma cell plasticity should be a new strategy to better control disease progression. Clearly at present, tumor cell plasticity appears to be one of the major obstacles for the cure of malignancies as it makes tumor cells highly adaptable to microenvironmental changes, enables their phenotype switching among different forms, and favors the generation of pro-metastatic cancer cell subsets. All of the events described can be triggered by hypoxic stress. Unlike genetic alterations, which directly modulate tumor cell function, hypoxia influences both tumor and stromal cells, shaping stromal reactivity and tumor vessels (although not discussed in detail here), while interfering with host immunity. More research is now needed to gain knowledge about the crosstalks at play between these components. Clearly, if the immune system plays the music, we believe that the microenvironmental hypoxic stress plays the tune. Therefore, targeting of the microenvironmental components to attenuate its hostility should provide new opportunities to adapt treatments for each individual, develop new combinatorial therapeutic strategies, while improving treatment efficacy. Given the potential of hypoxia to inhibit tumor promoting pathways in both stromal and malignant cells, the development of new agents inhibiting HIF signaling directly or its downstream effectors holds great promise for cancer immunotherapy with more integrative, efficient, and adaptive approaches.

## Author Contributions

SC directed numbers of the studies that are discussed in this Frontiers Mini Review. SB has significantly contributed to SC’s research work. SB and ST prepared Figure 1 and Figure 2, respectively. SC and ST wrote the manuscript and discussed concepts.

## Conflict of Interest Statement

The authors declare that the research was conducted in the absence of any commercial or financial relationships that could be construed as a potential conflict of interest.
